# Supplementary material for: The Growth–Climate Relationships of Three Dominant Subalpine Conifers on the Baima Snow Mountain in the Southeastern Tibetan Plateau
Source: Plants (Basel). 2024 Jun 14;13(12):1645. doi: 10.3390/plants13121645 (PMC11207451; doi:10.3390/plants13121645)

Figure S1 Annual trends of mean temperature and precipitation (1935–2019). The climate data were extracted from the grid point nearest to our sample site from the gridded Climatic Research Unit dataset (CRU TS4.07).

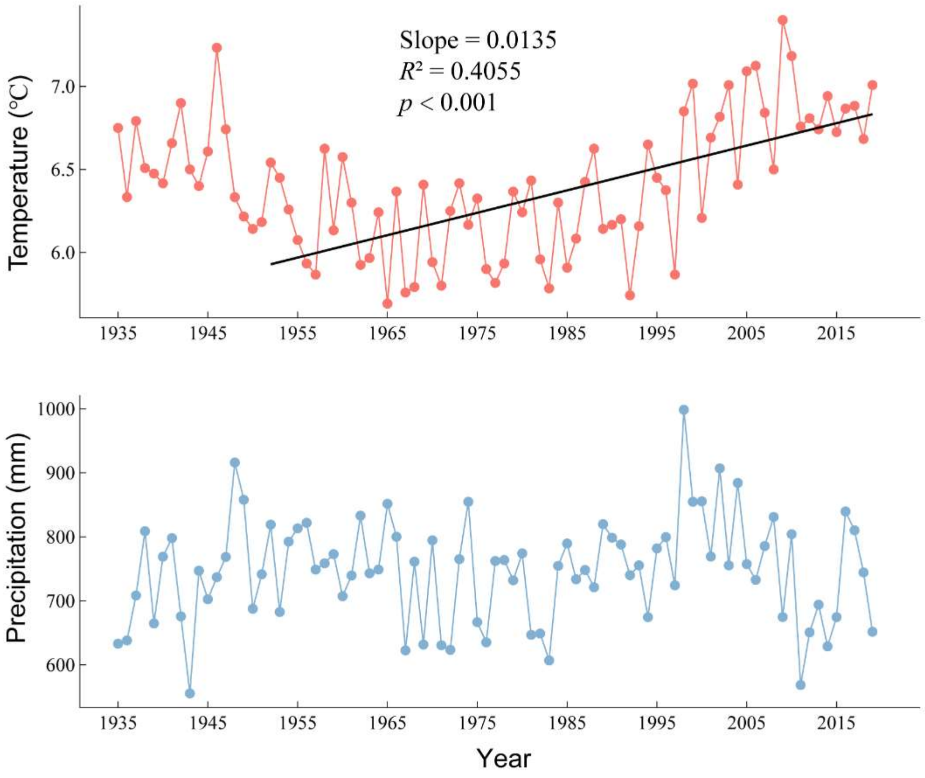

Figure S2 Diagnostic plots of the GAMM models, by species. (A) Residuals vs fitted values. (B) Observed basal area increment (BAI, log scale) vs fitted values. (C) Observed BAI (in mm^2) vs fitted BAI (in mm^2).

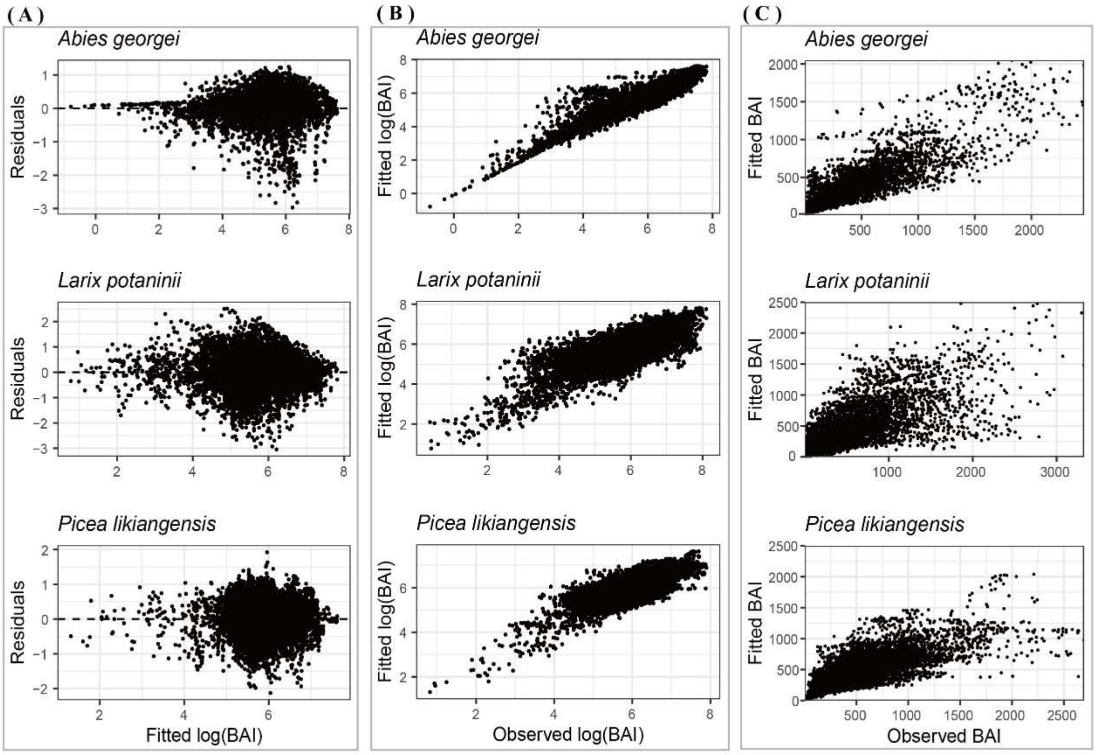

Figure S3 Basal area increment (BAI) and log (BAI) residuals chronologies for each species.

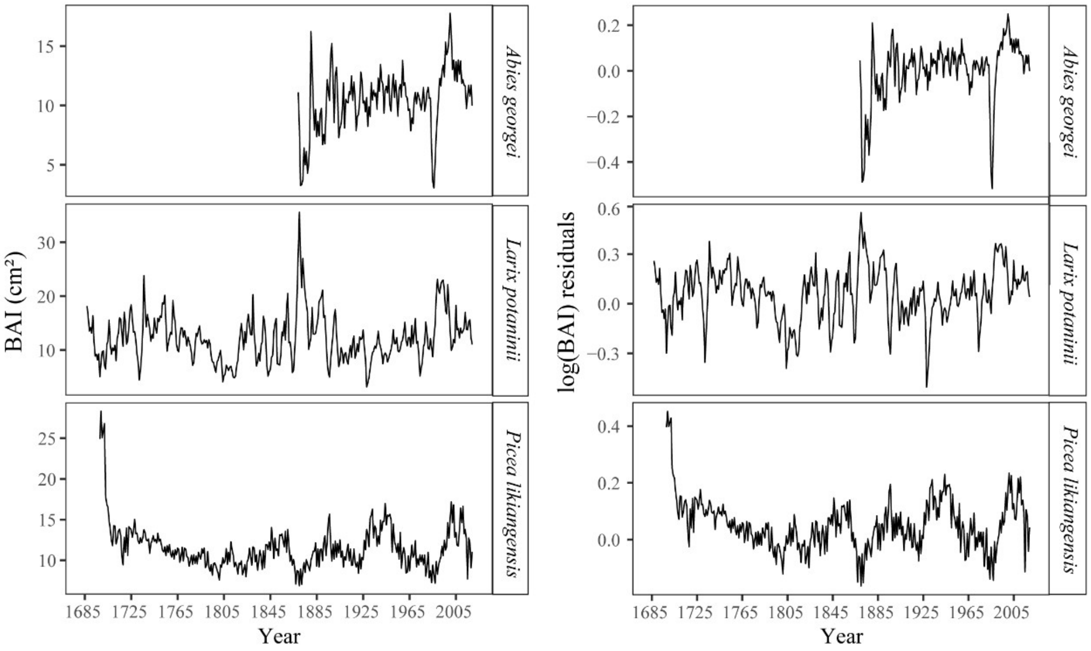

Supplement: Supplementary file 1 [file plants-13-01645-s001.zip › plants-3028957-supplementary.pdf]
